# Educator and student perspectives for enhancing international students’ sense of belonging in Ontario secondary schools

**DOI:** 10.1177/17454999251385720

**Published:** 2025-10-15

**Authors:** Clayton Smith, George Zhou, Thu Thi Kim Le, Atiya Razi, Michelle Marcuz, Chi Lam Chan, Thivya Sriramachandran

**Affiliations:** 1 University of Windsor, Canada; 2 441082 Ho Chi Minh City University of Technology and Education, Vietnam; 3 176145 Greater Essex County District School Board, Canada; 4 25809 The University of Hong Kong, Hong Kong

**Keywords:** international students, sense of belonging, Ontario secondary schools, Canadian education

## Abstract

This study explored the current educational practices and strategies secondary schools implement to enhance a sense of belonging for their international students for academic achievement, the development of teacher and peer relationships, and school commitment and involvement. The study applied a convergent mixed-methods approach, which incorporated both qualitative and quantitative elements that engaged 45 international students and nine teachers/staff. The combination of these two types of data shed light on six key themes: (a) school, teacher, staff and student connections; (b) peer relationships; (c) communities, clubs, and social groups; (d) classroom motivation and engagement; (e) available resources and school systems; and (f) sociodemographic variables, in enhancing international students’ sense of belonging. Recommendations for professional practice are discussed, along with implications for institutions, educators, and families.

## Introduction

The number of international students studying in Canada has been rising gradually, nearly 170% over the past decade (CBIE, 2018). Data indicates that 75% studied at the post-secondary level, 15% in primary or secondary school, and 10% at other levels. Despite the diverse educational levels, international students invest significantly in the opportunity to study abroad, including heightened cultural awareness, improved job prospects, and enhanced language skills (Le, 2024).

Recent studies highlight the link between achieving a sense of belonging and international students’ academic success (Hernández et al., 2016), mental and physical health (Slaten et al., 2016), and school retention (Masika and Jones, 2016). Sense of belonging is defined as a feeling of connection and importance or matters to others (Rosenberg and McCullough, 1981). Sense of belonging promotes positive affective states, supports healthy social and psychological development (OECD, 2015). It is also associated with lower dropout rates (Davis, 2020) and related to academic engagement and success (Sinacore and Lerner, 2013). Therefore, institutions should prioritize creating a welcoming community to enhance international students’ sense of belonging, ensuring academic achievement.

However, many international students do not feel welcome at their new schools, leading to difficulties in forming meaningful relationships, anxiety, and a lack of belonging (Chen and Zhou, 2019; Robinson et al., 2018). Several studies have identified barriers and effects to international students’ sense of belonging. Wolff (2014) identifies language barriers, challenges adapting to western education, limited intercultural experiences, and a lack of willingness among domestic students to force connection. Many international students cannot create meaningful relationships with domestic students, suffer from anxiety, and do not have a strong sense of belonging (Robinson et al., 2018). This absence of connection is tied to elevated depressive symptoms among international students, exceeding the average rate. While approximately 20%–25% of high school students report depressive symptoms in general (Centre for Addiction and Mental Health, 2023), 36.7% of international students reported such symptoms (Shadowen et al., 2019).

While existing research has tackled barriers faced by international students in higher education, a gap remains in understanding these barriers’ specific impact on sense of belonging in secondary education. This study addresses this gap by exploring educator and student perspectives for enhancing international students’ sense of belonging in Ontario secondary schools. The aim is to aid institutions in enhancing engagement, mental health, and academic performance among international students. The research intends to identify strategies that can improve international students’ sense of belonging upon their arrival in host countries. The following research questions guided our study.

(1) What are the educational practices being used to support achievement of international students’ sense of belonging in secondary-school educational environments?
(2) What are student perspectives and expectations regarding educational practices being used to support achievement of international students’ sense of belonging in secondary-school educational environments?
(3) What are educator perspectives and expectations regarding educational practices being used to support achievement of international students’ sense of belonging in secondary-school educational environments?

## Literature review

### Sense of belonging of international students

Sense of belonging at school is a psychological state in which the students “view schooling as essential to their long-term well-being, and this attitude is reflected in their participation in academic and non-academic pursuits” and results in “good relations with school staff and with other students; they feel that they belong at school” (Willms, 2003: p. 8). Attributes of countries, families, schools, teachers, and students are linked to international students’ sense of belonging at school. International students who enjoyed greater social or cultural communication with family and perceived stronger relationships with peers and teachers experienced greater sense of belonging (Chiu et al., 2016).

The students who have the attitude of not sharing this sense of belonging at school become disaffected from school (Jenkins, 1995), resulting in gradual disengagement from school activities and take part in disruptive behavior and exhibit negative attitudes towards teachers and peers (Willms, 2003). Students with increased sense of belonging have improved school engagement, academic performances, and academic success (Allen et al., 2021). There are numerous arguments claiming that the school environment has a strong effect on students’ involvement and sense of belonging. A sense of belonging at school is key to overall student development. Students who feel acceptance, respect, and inclusion in the school climate, achieve academic success and show positive social emotional outcomes (Allen et al., 2021). Students experience schooling during classes conducted by teachers, so teachers play a crucial role in fostering students’ sense of belonging (Anderman and Leake, 2007; Cemalcilar, 2010).

### Ways to enhance a sense of belonging

Many international students may find it difficult to integrate themselves into their new western school and community as they come from countries that have very different educational and cultural systems (Chen and Zhou, 2019; Le and Huyen-Nguyen, 2024). The following literature discusses ways that students, instructors, and schools can create a sense of belonging for their new students at the secondary level. The literature focuses on two main themes: encouraging teacher and peer relationships and encouraging school commitment and involvement.

#### Encouraging teacher and peer relationships

When international students first move to their new host country, a major struggle they face is isolation (Smith et al., 2019; Zhou and Zhang, 2014). Encouraging relationships between teachers and students can be an excellent way to decrease isolation and improve sense of belonging as it mediates the link between immigrant status and student engagement (Chiu et al., 2012). Students with immigrant backgrounds often face social and cultural barriers that can hinder their participation in school life, but positive interpersonal relationships have been shown to foster greater academic motivation, participation, and overall engagement (Chiu et al., 2012; Le, 2024). For teachers to create meaningful relationships with their students, they should first prepare themselves for diverse classrooms (OECD, 2015) which can be achieved by participating in staff development workshops. Teachers who are aware of international student needs can make appropriate changes in their classroom and teaching style, creating a sense of welcoming for students (Suarez-Orozko et al., 2010). From there, beneficial teacher relationships can emerge. With post-secondary students, there are different approaches that should be taken to create instructor relationships. Reaching out to international students where they regularly meet is a great way to create visibility for student affairs services; academic advisors can make onsite advising hours at these sites for example (Stebleton et al., 2010). These efforts can also show international students that professionals are accessible and friendly (Stebleton et al., 2010), which can hopefully increase comfort and belonging at their institution. Effective counseling programs have also been proven to increase a sense of belonging for international post-secondary students as it allows them to create a connection with someone. They can safely talk about different issues such as stress, anxiety and depression (Yan, 2020). Social media should be incorporated into these counseling sessions since it can be a facilitator for communication and creating relationships (Singh, 2018).

Among primary and secondary students, teacher support and encouragement are associated with higher academic achievement, which in turn fosters greater student engagement (Chiu et al., 2012). Teachers who demonstrate effective classroom discipline promote a sense of responsibility among students, further contributing to increased academic success, engagement, and a stronger sense of belonging (Chiu et al., 2012). Moreover, younger students tend to feel more comfortable and connected when they hear others speaking their native language; thus, instructors are encouraged to support the use of students’ first languages, as doing so affirms their cultural background and helps maintain ethnic identity (Asanova, 2005). The academic connections have been proven to help with adjustment in the host country (Hartman, 2009). Teachers need to be diligent and ensure that discrimination students face is kept at a minimum. This can be done by connecting with international students and understanding their relationships with domestic students (Suarez-Orozko et al., 2010).

Regarding peer relationships, the literature reports similar findings across for all age groups. Encouraging international students to build friendships with domestic students can foster a stronger sense of belonging as these relationships provide emotional support, schoolwork assistance, and opportunities to improve language skills (Chan et al., 2020). Although many students may feel more comfortable connecting with peers who share the same cultural or ethnic identity (Griffin and McIntosh, 2015), it is important to encourage friendly relationships between international and domestic students, as international student adjustment can be facilitated more effectively through interactions with host students (Hale et al., 2019).

#### School commitment and involvement

Hartman suggests that being a part of a group provides individuals with a sense of belonging (2009). He also shows that the stronger the grade point average, the greater the academic engagement (Sinacore and Lerner, 2013). Students with greater self-efficacy, self-concept, or reading achievement achieve a higher sense of belonging (Chiu et al., 2016). International students involving themselves in their school community and committing to the academics experience a positive self-concept and a sense of belonging in their new host countries (Hartman, 2009). One way to connect with the school community is by encouraging families to enroll their younger children in pre-primary education programs. To help with providing language instruction quickly, offering high quality childhood education, and allowing the students to familiarize themselves with western school norms (OECD, 2015). Providing primary school students educational resources at home helps them get more involved with their academics, increasing their sense of belonging (Chiu et al., 2012). Encouraging extracurricular involvement is also a key as it enhances language skills allowing international students to have greater success at connecting with domestic students and increasing comfort and belonging in the school community (Chan et al., 2020).

One of the first things that should be provided to secondary-school students is information on class rotation and the bell system (Suarez-Orozko et al., 2010). In many secondary schools, students move between different classrooms and teachers for each subject, following a structured timetable marked by bells that signal the beginning and end of each period. Understanding this system helps new students navigate the school day with greater ease and confidence. Many students may be coming from areas that do not use these methods so providing them with such information will ease their transition. Providing international students with different school resources helps them to become aware of their school community and encourage involvement. Introducing multicultural weeks allows international students to share their traditions to promote multicultural understanding and increase sense of belonging. Post-secondary undergraduate students also need some form of introduction to campus. Providing orientation for international students helps them become aware of their new community and make them more inclined to be a part of it (Yan, 2020). Orientation should include tours of health centers, diet and nutrition tips, advice to deal with homesickness and introduction to wellness programs (Yan, 2020) and explanation of college and teaching culture in higher education to help with fitting into the academic community (Stebleton et al., 2014).

Another way to create a connection on campus is to provide them with multicultural learning centers and advising offices to create a sense of community and belonging (Stebleton et al., 2014). Providing English courses to all international students can help language development and increase communication and lead to enhanced sense of belonging (Darwish, 2015). Some routes the institution can take to make students feel more involved and welcome on campus is by providing more scholarships. Financial struggles make it difficult to adapt and involve themselves on campus, so providing financial aid could reduce some of the stress and enjoy campus life (Rivas et al., 2019). Schools reduce the number of credits required per semester, so international students have more time to experience the campus in its entirety (Darwish, 2015). University of British Columbia has developed the Professional Development Program for International Teaching Assistants (PDITA) that focuses on student integration, cross-cultural communication, and effective teaching strategies, which creates a safe space where students could share their experiences as international students and many students thought of it as a safe space where they felt comfortable and were able to use it during the transition process (Guo and Chase, 2011).

## Theoretical framework

**Figure 1.**
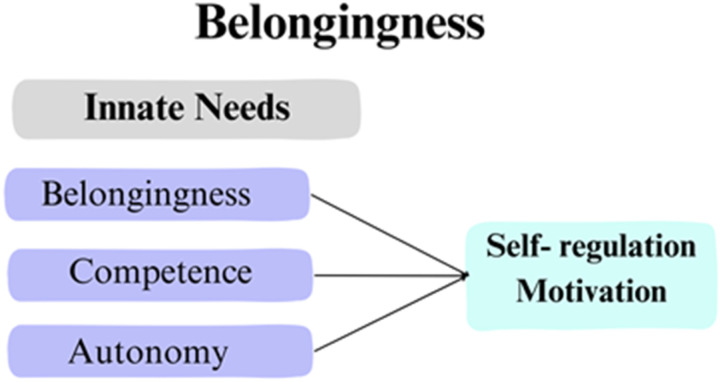
Baumeister and Leary’s (1995) Need-to-belong theoretical framework. *Note:* Adopted from self-determination theory and the facilitation of intrinsic motivation, social development, and well-being by Ryan and Deci (2000).

The current study is influenced by Baumeister and Leary’s (1995) need-to-belong theoretical framework (Figure 1), which suggests that the need to belong is a fundamental human motivation and that “a need to belong, that is, a need to form and maintain at least a minimum quantity of interpersonal relationships, is innately prepared (and hence nearly universal) among human beings” (p. 499). The absence of belongingness or experiencing social isolation can lead to negative psychological consequences, such as loneliness, depression, and anxiety. Baumeister and Leary suggest that group behavior and close relationships help individuals build a sense of belonging and prejudice of race, gender, and national origin from the in-group members will prevent newcomers from being involved in a new group. According to Ryan and Deci (2000), belongingness is one of three innate psychological needs that contribute to intrinsic motivation. A sense of autonomy, competence, and overall well-being can be experienced when people feel connected and accepted in their social environment. This framework explains and supports the motivational dynamics for international students to build a sustaining sense of belonging with peer students, teachers, and school staff.

**Figure 2.**
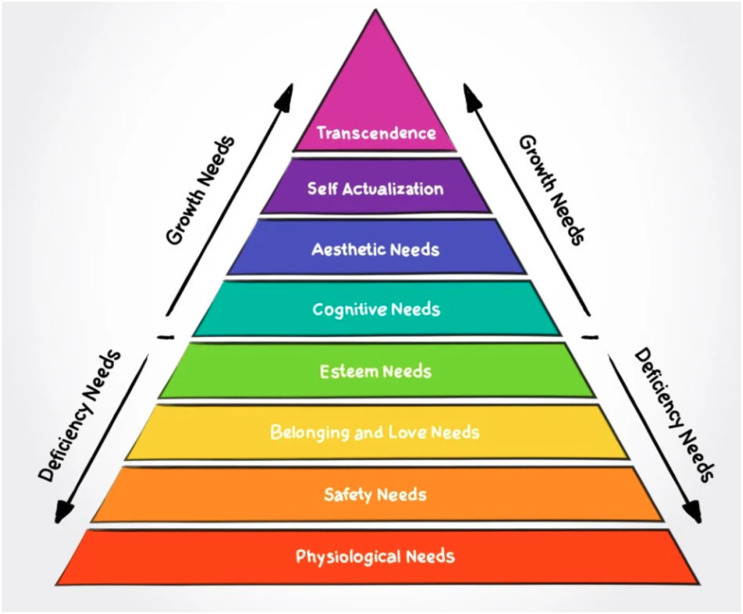
Maslow’s hierarchy of needs motivational theory (McLeod, 2024).

Another conceptual background that guided this research study is Maslow’s hierarchy of needs motivational theory (Figure 2) that claims human actions are motivated by a five-level hierarchy of physiological and psychological needs, from basic to complex (Maslow, 1943, 1954). Belonging and love were placed at the third most essential deficiency needs, compared to the top level of growth or being needs. Maslow (1962) highlights how the human need for belonging is the driving force behind motivating action. Identifying with a particular group or groups and having friendly and intimate relationships were viewed as essential human needs (Hale et al., 2019). Connectedness to friends or the community enhances resilience, while isolation leads to burnout (Hale et al., 2019). This theory provides a potential pathway for explaining how international students adapt to study and everyday life in a school context.

## Methodology

### A mixed-methods approach

**Figure 3.**
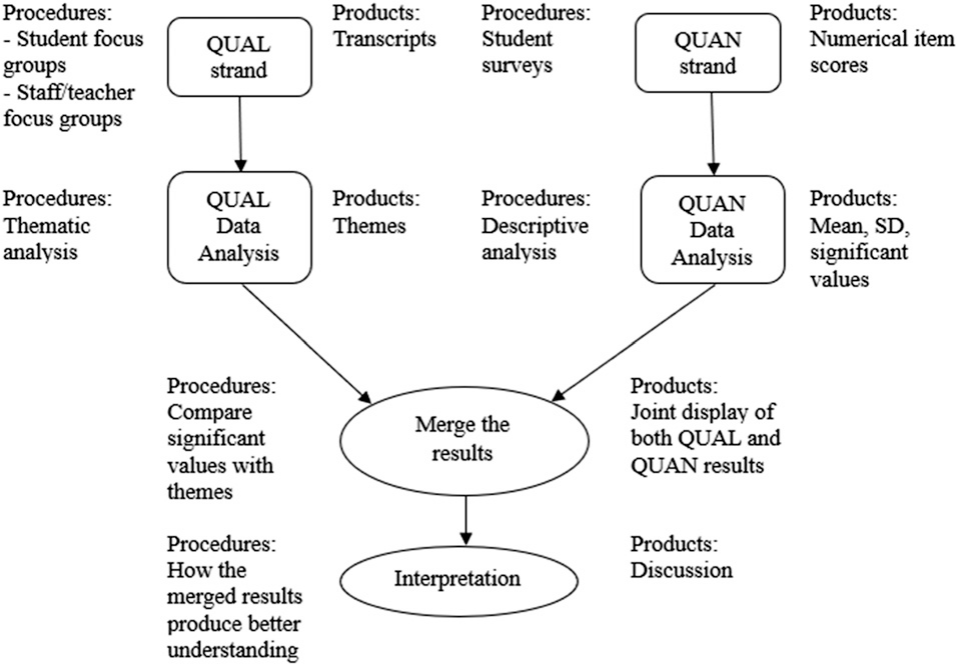
The procedure of a convergent mixed-methods design (Creswell and Plano Clark, 2018).

Mixed-methods research is defined as “research that involves collecting, analyzing, and interpreting quantitative and qualitative data in a single study or in a series of studies that investigate the same underlying phenomenon” (Leech and Onwuegbuzie, 2009: p. 267). This study applied convergent mixed-method research design (Creswell and Plano Clark, 2018) to collect both qualitative and quantitative data at the same time during the academic year of 2022–2023 (Figure 3). The fundamental objective of this approach is to validate one set of findings with the other and achieve a more comprehensive understanding of the research problem and questions (Creswell and Plano Clark, 2018). The quantitative and qualitative data are compared, combined, and integrated to obtain the synergy and strengths of both research methods. In this study, quantitative data was collected from student surveys, and qualitative data was obtained from student and teacher/staff focus group discussions to answer three research questions.

### Participants

This study included 54 participants from a Southwest Ontario public-school board: 45 international students and nine teachers and staff from the same secondary schools. In accordance with REB requirements, the survey did not collect students’ sociodemographic information to maintain anonymity. A subset of 14 international students participated in focus group discussions. These participants represented grades 10–12 and included both newly arrived and returning students from six different secondary schools within the board. Tables 1 and 2 provide the sociodemographic profile of the focus group participants.

**Table 1.** Focus group students’ sociodemographic information.

Pseudonyms	School	Grade	Status
IS-1	Westview	12	Returning
IS-2	Massey	10	New
IS-3	Massey	12	Returning
IS-4	Westview	10	Returning
IS-5	Westview	10	New
IS-6	Riverside	11	Returning
IS-7	Herman	12	Returning
IS-8	Tecumseh	12	Returning
IS-9	Herman	11	New
IS-10	Kennedy	11	New
IS-11	Herman	12	New
IS-12	Kennedy	12	New
IS-13	Kennedy	11	New
IS-14	Westview	10	New

**Table 2.** Focus group teachers and staff’s sociodemographic information.

Pseudonyms	Schools	Roles
T1	Massey	Guidance
T2	Massey	Guidance
T3	Massey	ELL
T4	Westview	CYW
T5	Massey	ELL
T6	Westview	Admin support
T8	Westview	CYW
T9	Massey	Admin support

### Recruitment procedures

The procedure started by recruiting students via the recruitment letter including a consent form sent to parents. This letter was translated into international students’ native languages by native speakers with professional English proficiency, to guarantee that both parents and students understand the purpose and the significance of the study. Staff/teachers also received the same letter with a consent form. Staff/teachers were asked to post the recruitment letter on their school and class Edsby pages. Edsby, a cloud-based tool that offers real-time access to student’s attendance, schedule, activities, and classroom work, was chosen as the recruitment platform because it is used as a communication platform that serves to provide both educational and community information for students and families. International students received an invitation email including a brief description of the research and a link to the consent form and the survey. In this study, students completed two online surveys: the first one at the beginning of the first semester and the second at the end of the second semester. They also participated in focus groups discussions twice per semester. Teachers/staff participated in one focus group each semester.

### Data collection

#### Survey

For the online survey, a consent form was sent to international students’ parents. This letter described the goal of the study, the principle of voluntary participation, the right to withdraw from the study at any time without consequence, and the contact information from the school board. No personal information was collected through the online system. The survey, based on the Psychological Sense of School Membership Scale, developed by Goodenow (1993), includes the five-point Likert scale with 43 question items asking factors and aspects from the schools and life in Canada which can influence their sense of belonging. The five-point Likert scale ranges from 1 = Not true at all to 5 = Completely true. At the end of the survey, the students were asked if they were willing to participate in the prize draw when the study was completed. Surveys were administered (pre and post) at the beginning and end of the 2022–23 academic year.

#### Focus group discussions

Student focus group discussions were conducted twice each semester via Microsoft Teams, with both new and returning students attending the one-hour discussions. Staff/teachers attended one hour-long focus group discussion per semester. The discussions were voice recorded with a recording device to ensure their privacy and confidentiality were highly respected. The questions for both the staff and students focus group were drawn from the literature review, and discussed and reviewed by the research team, including the principal investigator, co-researchers, representatives from the school board, and graduate students. Question topics included student and staff/faculty perceptions on the welcoming environment, peer interactions, classroom experience challenges, assessments, group work, class participation, and overall sense of belonging.

### Data analysis

Quantitative data from students’ surveys were analyzed by the IBM SPSS version 25. Standard descriptive analysis was run to calculate the mean of the survey items. Qualitative data from students and staff/teacher focus group discussions were analyzed by reflexive thematic analysis (Braun and Clarke, 2012) using MaxQDA version 24. The process begins with data familiarization, where researchers read and re-read the transcripts. Next, initial codes were generated to identify meaningful data segments based on the key points from literature review. If new codes emerged, they were grouped into themes. All these codes were then organized into potential themes. These themes were reviewed by the whole team, ensuring that they were accurately analyzed and interpreted. The aim of the thematic review was to capture significant patterns across the data, and to answer the research questions. This is an iterative and flexible process, allowing the researchers to engage deeply with the data and enhancing the accuracy and depth of the data analysis step. The results from quantitative and qualitative analysis were finally compared and merged to draw a comprehensive picture of educator and student perspectives for enhancing international students’ sense of belonging in Ontario secondary schools.

### Validity and reliability

The value of the Cronbach alpha, frequently referred to as the alpha coefficient of reliability, provides a coefficient of inter-item correlations, that is, the correlation of each item with the sum of all the other relevant items and is useful for multi-item scales (Cohen et al., 2018). The Cronback’s Alpha value of the adopted survey was calculated in previous research at 0.84 (Alkan, 2016). All focus group questions were developed from the literature review and guided by the theoretical framework. All these questions were also collectively reviewed by our 12-member research team including representatives from the University of Windsor and the Greater Essex County District School Board.

## Results

### Survey findings

Survey findings identify the level of belonging experienced by international students at the studied secondary schools. Two surveys were conducted to measure this level: one in the first semester and another at the end of the academic year. The surveys utilized a 5-point Likert scale, where higher scores indicate a stronger sense of belonging. The results show a notable increase in their sense of belonging as students progressed through their studies, with the initial survey’s mean score of 3.25 and the second one at 3.95. This positive change suggests that international students at Ontario secondary schools feel reasonably connected and integrated during their first semester; then strengthens their belonging over the course of the academic year. Several factors contributed to this improvement, such as increased familiarity with the school environment and culture over time, improved language and communication skills, effective support systems provided by the school, greater involvement in school and extracurricular activities, or social networking and friendship development. These factors will be analyzed and discussed further in the next sections. Results of 10 selected survey items with mean scores exceeding 3.50 on a 5.00 scale (Table 3), indicate a generally positive perception among the students. The highest scores are observed in items related to respect and friendliness from teachers, with “Teachers at my school respect me” and “People at my school are friendly to me” both receiving a mean score of 4.43 and 4.00, respectively. These results suggest that students feel respected by their teachers and perceive their school environment as friendly. Additionally, the items “People at my school notice when I am good at something” and “I am treated with as much respect as other students in my school” both scored 3.86, highlighting that students feel acknowledged and treated equally.

**Table 3.** Selected items with mean scores over 3.50.

Selected items with mean scores exceeding 3.50/5.00	Mean
Teachers at my school respect me.	4.43
People at my school are friendly to me.	4.00
Teachers at my school know that I can do good work	4.00
People at my school notice when I am good at something.	3.86
I am treated with as much respect as other students in my school.	3.86
Most teachers at my school are interested in me.	3.71
Other students at my school like me the way I am.	3.71
On most days, my group works well together.	3.67
I feel like I belong to my school	3.57
There is at least one teacher or adult I can talk to in my school if I have a problem.	3.57

The item *“Other students at my school like me the way I am”* received a mean score of 3.71, indicating that students feel accepted by their domestic peers. This welcoming environment can foster a stronger sense of belonging for international students, as inclusion often starts with domestic students who are confident and friendly toward others (Rowan et al., 2021). The sense of belonging, measured by the item “I feel like I belong to my school,” received a mean score of 3.57. While positive, this score is lower than other aspects such as respect and teacher interest. This suggests that while students generally feel respected and acknowledged, their overall sense of belonging may still need further support to reach the levels of other positive experiences within the school environment.

### Findings from student and staff/teacher focus group discussions

After coding all the student and staff/teacher focus group transcripts, we applied thematic analysis (Braun and Clarke, 2012) to interpret and identify the data into the four key themes: (a) school, teacher and staff support, (b) classroom practices and teaching pedagogies, (c) clubs and extracurricular activities, and (d) student mentors’ program.

#### Theme one: School, teacher and counselor support

Findings from teacher/staff data showed that when international students first came to the schools, school, staff, and teachers make every effort to make them feel welcome in the new learning and living environment. Two teachers (T1 and T2) indicated, “we have the guidance of office and staff counselors who can answer any questions that students are unsure of” and “these supports make students feel comfortable to talk with.” Some students found the resources provided by the schools useful. For instance, IS-7 mentioned they had the CYW (child youth worker), CYF (change your future) advisors, and teachers at school to provide emotional and academic support. IS- 4 expressed his appreciation for the guidance counselor for providing information about courses and credits: “the first day I came to the school. I talked with the school counselor, and the way he talked to me was not too rushed, and he just talked to me calmly and he provided me with necessary information.”

Teachers can also be identified as a source of support for international students’ sense of belonging, as one teacher shared that “we have teachers always outside welcoming them into their classrooms as they come in the morning.” From students’ points of view, teachers’ assistance is found to be helpful in enhancing their sense of belonging. Most students indicated that their teachers were amiable in offering help and creating a welcoming environment. IS-1 shared that “teachers are super kind and nice to us. They ask me, ‘How *are you doing these days?’ Do you have any problems with your course?”* IS-4 also expressed that teachers made time to share with her some Canadian cultures, and she also shared her culture with the teachers. All international students appreciated the effort their teachers put into supporting them.

However, IS-3 expressed that the different teaching styles in Canada and his home country had created some challenges in his first semester in Canada:

When I ask for some support from the teacher, I must go to ask them myself. But in China, it’s usually the teacher who will come to ask every student if they don't understand. I started to learn the differences in school systems. I ask the teacher more, so then I can get support.

This may indicate the need for teachers to take a more active approach for international students who are used to passive learning in their home countries to ensure enough support is given to them.

#### Theme two: Classroom practices and teaching pedagogies

Most international students believe group work helps them to make friends and allows them to interact and connect with other students, especially domestic ones. IS-2 mentioned that “it was very good for me to get to know more people. And I feel really good to be a member of the group. They ask me if I have some ideas, and I tell them my ideas.” Furthermore, some students expressed a sense of esteem and confidence when their group mates listened and respected their opinions and treated them fairly.

However, IS-3 shared that language difficulties prevented him from interacting with his domestic group members since he could not keep up with their pace. According to these examples, international students may not be able to benefit from group work due to long adaptation times and language barriers.

Nevertheless, some participating students showed concern about the work distribution in group work. IS-4 mentioned that sometimes only a few people would contribute to their group work. IS-1 also said “some international students are not that hard working. So, if I [am] to work with them, I’m helping them to improve their mark.” Other international students explained they needed time to learn and adapt to the working style. They might not contribute much to group work at first, but they try to help more once they get used to it. IS-3 mentioned that language could play a role in affecting work distribution too because language difficulty reduced the chance to interact with his group mates as he could not follow their pace. These examples suggest that long adaptation time and language barriers can hinder the benefits group work brings to international students in building a sense of belonging.

The level of classroom engagement may also increase international students’ sense of belonging. Some student participants mentioned that the way their teachers taught in class made them feel more motivated to learn. For instance, IS-1 said “the teachers teach me why I need mathematics skills, and how I apply it in my life. I like studying in Canada because I know more about why and how instead of what.” Moreover, tasks and quizzes students complete in class can engage students. These assessments help them understand the knowledge better and make them feel motivated in learning, enhancing their sense of belonging to the class. As IS-7 indicated:

Here knowing that your coursework, your collaboration with others, your behaviour, your attitude, like even the smallest assignment that you do affects it (university entrance). You know you're not doing this for nothing. You're not doing this to write a big exam on the last day of the school. It's for really learning something.

#### Theme three: Clubs and extracurricular activities

International students’ sense of belonging is substantially enhanced via different extracurricular activities, such as sports and games, as T4 mentioned:

We started to notice that getting both current and new students into clubs, sports, etc. creates that sense of belonging. It gives them that willingness to achieve more. And we notice that through that we also have empowered the cultures for multiculturalism.

Clubs and other programs organized by domestic students at schools, such as Ambassador Club, Youth Ambassador, Freedom Academy, and EFL support programs, also support new students’ sense of belonging. A teacher mentioned that these clubs provide opportunities for students to contribute to the schools and clubs, which enhanced their sense of community and belonging:

We open Freedom Academy with lots of activities and spaces where international students can have a safe, inclusive, and interactive student-led environment. We want to make sure that they can have opportunities to develop a sense of belonging.

Youth Ambassadors invite schools from all over Ontario to come in and to share ideas and opinions with other schools. This creates leadership skills and opportunities for many students, makes liaison between the local community and our new students and international students.

Similarly, all international student participants indicated that school activities help them build more friendships with domestic students and achieve a sense of belonging in Canada. For instance, when being asked what improves the sense of belonging of the international student the most, IS-4 answered, “whenever there’s a school activity, a lot of students are engaging together and then we build a memory.” Other students also expressed the wish to make friends by joining different school activities. IS-1 mentioned that it could be helpful to use her native tongue in the club to communicate with other co-national students when she did not want to talk in English. IS-4 also indicated that she felt more comfortable with the international students as they all have the same feeling of being away from home. Because of the similarities that international students share, it is easier for them to get along. Domestic students who participate in these activities also play a key role by welcoming international peers and creating opportunities for genuine cross-cultural friendships. The international students club serves as a platform to bring these students together and provides a space for them to share their feelings and experience in a new country. As a result, they form a small community and build a sense of belonging to this community.

The school clubs and activities help support student emotional, psychological, and overall well-being. However, some student participants said their schools do not offer a lot of clubs and activities that engage them and make them feel connected. For instance, according to IS- 2, “If there are more activities that I like, I will try, but there aren’t so I can’t.” The limited number of school activities that interest international students may impede the opportunity for these students to establish a sense of community.

#### Theme four: Student mentors’ programs

Student mentors, including both local and other international students, take responsibilities to help newcomers or brand-new international students via various activities, such as English support and campus tour. As T9 said:

We connect new students with other students who are sort of mentors for that first bit until they get to know other students. For these new kids, it's always so much nicer for another student to take them to their lockers and show them where that is and show them the cafeteria and escort them to classes.

Some student participants indicated that providing enough information regarding the school systems, classroom locations, and resources available at the beginning of their study would help them better adapt to the new place. SI-1 mentioned that she only knew her school had a library 1 week after she started school. She also suggested that it would be helpful if someone could give her a school tour and introduce the school facilities and teachers to her. Some student participants indicated that the schools provided them with maps of the campus when they first arrived. However, only providing school maps may not fully fulfill international students’ needs to know the school better. For instance, IS-2 said that he found the map confusing because he did not know the purpose of the lockers on the map, as there were no lockers at schools in his home country.

Nevertheless, teachers argued that students’ sense of belonging depends on their individual variables, such as prior experience, personality traits, grade levels, and length of staying in a foreign country. T9 said that “I find that our short-term students, the students especially that are here maybe just for a semester or a 3-month period and so on, it’s more of an experiential learning opportunity versus those who are here to get credits.” T5 also indicated “there is one student who was bullied and being really low on the academic ladder in their home country. When he is here, he tries to not only adjust to the new system but also to figure it out.”

## Discussion

The integration of quantitative and qualitative data in this mixed-methods study provided a comprehensive understanding of educator and student perspectives on international students’ sense of belonging in secondary schools. This methodological approach allowed for triangulation of findings and deeper insights into the complex factors that influence belonging among international students. Six themes emerged from the analysis, encompassing the multifaceted nature of belonging experiences: (a) school, teacher, staff and student connections; (b) peer relationships; (c) communities, clubs, and social groups; (d) classroom motivation and engagement; (e) available resources and school systems; and (f) sociodemographic variables. These themes collectively illustrate the interconnected social, academic, and institutional factors that shape international students’ sense of belonging in their school environments. The findings align with established theoretical frameworks, particularly Baumeister and Leary’s (1995) belongingness hypothesis, which emphasizes that humans have a fundamental need for positive, stable relationships and group membership. Similarly, Maslow’s hierarchy of needs theory provides a complementary lens for understanding how belonging serves as a prerequisite for academic achievement and personal growth among international students in secondary school settings.

### School, teacher, staff and student connection

Regarding non-academic aspects, this study underscores the significance of interpersonal connections for enhancing international students’ sense of belonging. Student participants reported that their teachers warmly welcome them and readily address their questions. The warm welcome and ongoing support from teachers and staff reflect Baumeister and Leary’s idea that positive interpersonal interactions are essential for fulfilling their need to belong. Simple gestures like checking in on their well-being and engaging in casual conversations significantly contribute to their sense of worth. This result aligns with the previous study suggesting a robust correlation between teacher–student relationships and students’ sense of belonging at school (Chiu et al., 2016). Therefore, it is imperative for educators to foster an inclusive environment and cultivate strong teacher–student relationships, enhancing a greater sense of affinity with the new environment. Moreover, students from countries with passive learning systems may not actively connect with teachers. Hence, teachers/staff should proactively initiate efforts to build connections, ensuring even reserved students could develop close bonds with the instructors.

Most teachers also agreed that students feel exceptionally welcome as they embark on their new journey. The school, staff, and student mentors offer comprehensive orientation programs prior to formal classes, including school tours and meetings with teachers. These initiatives enable students to familiarize themselves with their instructors, establish contacts for inquiries, and identify their initial class schedules.

Additionally, teachers/staff contribute to international students’ sense of belonging through various forms of social support. Counselors address various concerns, while teachers offer day-to-day assistance, share personal stories, and bolster understanding during lessons. These practices all contribute to stronger connections between teachers/staff and students, fostering a heightened feeling of belonging among international students.

### Peer relationships

Apart from the teacher–student connection, peer relationships are crucial for increasing students’ sense of belonging. Teachers observed that international students rapidly form supportive friendships due to shared languages, backgrounds, and experiences in Canada. Schools pair newcomers with mentors, often fellow nationals, but also introduce them to local and other international students for a broader perspective and inclusion. These bonds create a sense of community outside academics, aligning with previous research highlighting cultural understanding and shared experiences as facilitators of close connections (Gomes, 2020; Li and Zizzi, 2018; Rivas et al., 2019). Likewise, Baumeister and Leary’s need to belong theory highlights how forming supportive friendships and participating in peer mentorship meets the fundamental need for positive interpersonal relationships. These connections help students feel valued and integrated into their new environment. From Maslow’s perspective, these peer interactions address the love and belonging level of his hierarchy, fulfilling students’ need for social acceptance and emotional support.

Teachers and staff encouraged students to take on leadership roles to aid peers from minority backgrounds, along with the support of school staff, teachers, and friends. These findings have been shown in previous studies to significantly assist international students in adapting and feeling more connected (Iorga et al., 2020; Xiong and Zhou, 2018).

In addition, collaborative group work serves as an educational practice fostering peer interaction. Some students find this practice particularly effective in building local friendships, aligning with research indicating that more local friendships correlate with greater satisfaction and reduced homesickness (Hendrickson et al., 2011). However, adapting to host country group work dynamics might be challenging, especially for those with language barriers hindering communication with domestic students. Empirical studies show that language barriers impede international students’ interaction and communication with domestic students (Cena et al., 2021; Smith and Khawaja, 2011). As a result, teachers should offer clear group work guidelines and additional language support, facilitating participation and effective communication within groups.

### Communities, clubs, and social groups

International students indicated that participating in school activities is key for them to feel a sense of belonging. Events like dress down days or Valentine’s Day candy exchanges foster a collective atmosphere where everyone, regardless of background, participates together, promoting a strong sense of community and belonging. Engaging in extracurricular activities increases international students’ sense of belonging. Teachers and staff also expressed that clubs and programs, including sports and games, substantially provide ongoing opportunities for involvement and connection. These activities bridge the gap between international and local students. This point is congruent with conclusions from previous research highlighting the role of leisure activities (Gómez et al., 2014), social events (Zhou et al., 2018), and sports (Lee et al., 2019) in forming strong social networks and fostering a sense of belonging.

Furthermore, Baumeister and Leary’s need to belong theory emphasizes that engaging in communal activities, such as school events and clubs, helps fulfill the fundamental need for social connections and acceptance. These activities not only provide a shared space for interaction but also create a supportive network, addressing students’ need for belonging. From Maslow’s perspective, these interactions cater to the love and belonging level of the hierarchy, reinforcing international students’ integration into their new environment and enhancing their emotional well-being through meaningful social participation.

It is worthwhile to point out that beyond supporting international students’ adaptation, the cross-cultural interactions also enrich the experiences of domestic students. The presence of international peers provides local students with everyday opportunities to develop intercultural competence and global perspectives, an approach often described in the literature as internationalization at home. Domestic students gain valuable intercultural skills and broadened worldviews through sustained engagement with international peers (Beelen and Jones, 2015). Rowan et al. (2021) likewise note that local students can help bridge the gap between “longing and belonging,” while simultaneously enhancing their own sense of global connectedness. Emphasizing programming that fosters these mutual benefits can therefore strengthen the entire school community, not only by supporting international students but also by cultivating globally minded citizens among domestic students.

### Classroom motivation and engagement

There are some educational practices aiding student belonging but were not mentioned by teachers/staff. Classroom engagement levels are among these practices. Students’ classroom engagement influences their belongingness to classes. Canadian teaching methods, like focusing on mastery over grades and using diverse activities for learning reinforcement, increase student engagement compared to more teacher-centric, grade-focused approaches in their home countries. Students indicated that these instructional and engaging strategies foster engagement and build a sense of class belonging. This aligns with Osterman’s study (2010), highlighting that emphasizing mastery learning and engaging instructions instead of academic outcomes, stimulate students’ interest in learning, and elevate student belonging.

### Available resources and school systems

Providing comprehensive information about school systems and resources is another valuable practice desired by international students, although it was not mentioned by teachers/staff. When international students first arrive in a new country and a new school, they may not be familiar with how systems work in the school. Therefore, instead of just providing school maps, campus guided tours can alleviate their initial unfamiliarity and offer detailed explanations, especially aspects unfamiliar to the international students like locker use, ESL classroom location, and available academic and emotional support resources. Providing essential information online or in print for future reference also helps to prevent overwhelming students (Yan, 2020).

### Other demographic variables

Both teachers and students acknowledged that the sense of belonging varies among students. Having pre-existing friendships, shared backgrounds, and language fluency fosters a greater sense of connection. Besides, personality traits influence community adaptation. Even though being with other co-nationals, some students are not confident, shy, and less interactive with classmates in group work. They feel difficulties expressing and presenting ideas and are afraid of making mistakes when speaking English. All of these impede their ability to adapt to a new environment. These individual factors impact cross-cultural adjustment, as seen in studies by Le (2022). They concluded that international students with an adaptive personality or extroverted traits find it easier to be involved in a new culture since they can find solutions to daily issues. In the same vein, Yakunina et al. (2013) highlighted that it is quicker for those who possess universal-diverse orientation to make new friends with host nationals and to adapt to new values and cultures.

All teachers agreed that for international students moving to a new country is challenging, with homesickness, loneliness, and unfamiliarity. However, as students increase their participation in extracurricular activities, become more familiar with their environment, and improve their language skills, they gradually fulfill their need for belonging as described by Baumeister and Leary’s theory. Bastien et al. (2018) and Johnson et al. (2018) validate this shift from initial challenges to eventual comfort. These advancements allow them to forge deeper connections and feel more integrated into their new community. From Maslow’s perspective, this progression aligns with the fulfillment of the love and belonging level of the hierarchy, as students enhance social interactions and improve English proficiency, helping them establish a stronger sense of community and self-worth, ultimately facilitating their academic and personal growth.

Teachers emphasized that language proficiency also plays a role, granting independence and deeper connections. Improved English allows better communication with peers and teachers, fostering a stronger sense of belonging. Some students even transition from associating mainly with home nationals to engaging with local and multinational peers, reflecting increased cultural exploration. Similar findings by Xiao et al. (2019) highlight that longer stays lead to improved linguistic and cultural knowledge, heightening students’ sense of belonging.

## Conclusion

This study reveals that international students experienced an increase in their sense of belonging as they progressed through their studies. This suggests that international students at Ontario secondary schools feel reasonably connected and integrated during their first semester and perceive their sense of belonging strengthening over the course of the academic year. However, student-reported sense of belonging was rated lower than expected, which indicates the need for more sustained support for international students.

By identifying four key themes, the study underscores specific areas where interventions can be targeted to enhance international students’ sense of belonging. The first theme revolves around the pivotal role of interpersonal connections between international students with school teachers and staff. Teachers and staff who engage themselves with international students personally, psychologically, and educationally can significantly contribute to students’ sense of belonging. The second and third themes center on the significance of peer relationships and how to help international students to build such relationships. Peer connections through group work, clubs, and extracurricular activities offer great opportunities for cross-cultural friendship making. These collective events offer an inclusive platform for students to connect and develop social networks, shared interests, and a sense of community. The fourth theme emphasizes the role of active classroom engagement, instructional strategies, and accessible resources as integral to enhancing international students’ sense of belonging.

This study’s findings provide valuable insights for educators and institutions aiming to foster an inclusive and supportive environment for international students. Besides school teacher and staff, domestic students also shape the school climate in powerful ways. When local peers actively welcome and engage with international classmates, they help bridge the gap between “longing and belonging,” fostering cross-cultural friendships and a deeper, more lasting sense of inclusion for newcomers (Rowan et al., 2021). Strengthening these peer relationships can therefore be just as critical as teacher support in sustaining international students’ sense of belonging. It is crucial to develop strong interpersonal connections between school/staff/teacher and international students, or international students and other students, where they feel valued and supported in their transition to new countries. Improving English skills contributes significantly to international students’ sense of independence and belonging, enabling effective communication with peers and teachers and leading to closer connections and engagement. Cross-cultural learning opportunities should be provided as international students benefit from opportunities to attend different school activities/clubs/events and engage with local students, promoting cultural understanding and bridging the gap between diverse student populations. These key takeaways emphasize the complex and interconnected components influencing international students’ sense of belonging in Ontario secondary schools, offering guidance for educators, institutions, and families to create supportive environments for successful student adaptation into the school community.

While this mixed-methods study offers valuable insights into the ways Ontario secondary schools can enhance international students’ sense of belonging, several limitations should be noted. First, although the study utilized both quantitative surveys and qualitative focus groups to explore diverse perspectives, the findings were not disaggregated by students’ national and cultural backgrounds. As a result, the nuanced differences in experiences among students from varying backgrounds may have been overlooked. While disaggregation by subgroups could have compromised statistical power, future research should consider larger and more targeted samples to better understand how sense of belonging may differ across student populations (Suarez-Orozko et al., 2010). Second, the data were drawn from a limited number of schools, and participation was voluntary. This may have introduced self-selection bias, as students and educators who chose to participate could be those who are more engaged or have particularly strong (positive or negative) experiences with school. Therefore, the results may not fully represent the broader international student population in Ontario secondary schools.
